# Arterial Hypertension and Its Consequences Are the Main Predictors of Embolic Stroke of Undetermined Source

**DOI:** 10.1155/2023/3469755

**Published:** 2023-11-16

**Authors:** Alexander B. Berdalin, Daria D. Namestnikova, Elvira A. Cherkashova, Denis A. Golovin, Ilya L. Gubskiy, Vladimir G. Lelyuk

**Affiliations:** ^1^Federal Center of Brain Research and Neurotechnologies, Federal Medical Biological Agency, Moscow 117513, Russia; ^2^Pirogov Russian National Research Medical University, Ministry of Healthcare of the Russian Federation, Moscow 117977, Russia

## Abstract

**Methods:**

We performed a hospital-based prospective cohort study with 1,317 enrolled participants. We compared patients and healthy volunteers according to the main demographic, anthropometric parameters, stroke risk factors, comorbidities, and data of clinical and instrumental examination. In order to balance the study and the control groups for age and sex, the propensity score matching was performed. In order to generate the overall predictive model, a multivariate analysis was performed using the binary logistic regression method.

**Results:**

The following predictors of ESUS were identified in current study: arterial hypertension (AH); increased heart rate and pulmonary arterial systolic pressure (PASP); the presence of conduction disturbance; the enlargement of left, right atrium, and left ventricle end-systolic length; increased intima–media thickness (IMT) in right and left common carotid artery (CCA); lowered Montreal Cognitive Assessment (MoСA) cognitive scale score; the presence of subcortical microbleeds; central brain atrophy; the larger size of third ventricle; and the higher medial temporal lobe atrophy (MTA) score. The following risk factors were included in the final predictive model: the presence of AH (*p* < 0.0005; OR = 12.98 (95% CI: 4.53–37.21)) and PASP (*p*=0.018; OR = 1.13 (95% CI: 1.02–1.25)) and male sex (*p*=0.046; OR = 2.771 (95% CI: 1.017–7.555)). The Nagelkerke's pseudo-R-squared value was 0.404 and the significance of the Hosmer–Lemeshow test was 0.733, which indicate the goodness of the final logistic regression model.

**Conclusions:**

We propose that AH and its consequences are the main predictors of ESUS. The results of this study emphasize the importance of AH control for primary and secondary prevention of ESUS.

## 1. Introduction

Nowadays, ischemic stroke is considered to be one of the leading causes of death and long-term disability worldwide [1, 2]. Despite recent technological advances in clinical diagnostics and neuroimaging, in approximately one-third of cases, the exact mechanisms and etiology of cerebral ischemia remain unclear [3, 4]. This type of stroke of unknown origin is also called “cryptogenic” [5]. According to the most widely used TOAST classification of etiological subtypes of stroke [6], the criteria of cryptogenic stroke are as follows: two or more competing causes of stroke are identified, the patient is not sufficiently examined, and stroke etiology can not be determined despite extensive workup. Thus, cryptogenic stroke represents a heterogeneous group of ischemic strokes with variable etiologies, which makes it difficult to select appropriate secondary prevention [7, 8]. More recently, in 2014, the concept of “embolic stroke of undetermined source (ESUS)” was suggested [9]. ESUS is considered to be a subcategory of ischemic cryptogenic stroke that can be defined as nonlacunar infarction (ischemic lesion ≤1.5 cm according to neuroimaging) without significant (>50%) extracranial or intracranial atherosclerosis, major risk of cardioembolism, and the other identified specific causes of stroke [10]. The frequency of ESUS ranges from 7% to 42% according to the results of different clinical trials [10]. Some studies reported that ESUS is more frequently occurred in relatively young patients [10–13].

Different potential embolic sources of ESUS have been suggested and among them the most significant are cardioembolism (undetected paroxysmal atrial fibrillation, atrial and ventricular dysfunction or cardiopathy, silent myocardial infarction) and large artery atherosclerosis (nonstenotic arterial plaques, aortic arch atherosclerosis), and other causes (nonatherosclerotic arteriopathies, hypercoagulability disorders, etc.) [14–16].

Most commonly prescribed secondary prevention of ESUS includes antiplatelet, antihypertensive, and hypolipidemic drugs; however, patients receiving such a therapy have recurrent stroke rate 4%–5% per year [10]. Since cerebral embolism is believed to be the most likely stroke mechanism in ESUS patients, it was hypothesized that oral anticoagulation therapy may be more reasonable than antiplatelet therapy for secondary prevention. Unfortunately, randomized control trials showed that rivaroxaban and dabigatran are not more effective than aspirin in recurrent stroke prevention and, moreover, were associated with a higher risk of bleedings [17–20]. The reason for insufficient effectiveness of anticoagulation treatment is unclear and may be due to heterogeneity of ESUS [21]. Taking these facts into consideration, the etiology, pathophysiological features, and risk factors of ESUS remain not fully understood and require further investigations.

The main aim of the present study was to identify the most prominent risk factors of the ESUS and to generate the overall predictive model.

## 2. Materials and Methods

### 2.1. Subjects

The study was conducted in accordance with the Declaration of Helsinki and approved by the Institutional Ethics Committee of “Federal Centre of Brain Research and Neurotechnologies” of the Federal Medical Biological Agency of Russia. Individual informed consent was obtained from all the participants. A total number of 1,317 participants were enrolled in a hospital-based prospective cohort study: 1,171 patients with ischemic stroke admitted to the hospital from September 2019 to May 2021 and 146 asymptomatic volunteers (control group) without medical history of stroke or transient ischemic attack. For the group of patients with stroke, the inclusion criteria were as follows: less than 1 year from the onset of ischemic stroke; 3 points on the Rankin scale of the functional neurologic disability. The exclusion criteria were as follows: the presence of contraindications to perform a magnetic resonance imaging (MRI) study, the presence of oncological disorders, and severe general patient's condition. Subsequently, from the overall number of patients with ischemic stroke, 127 cases (10.8% from the total number of patients) of stroke of unidentified sources (cryptogenic) were selected after extensive workup. In the study group, we included patients with nonlacunar stroke, without signs of large vessel occlusion, significant extracranial and intracranial atherosclerosis (less than 50%), major risk of cardioembolic conditions (atrial fibrillation, left ventricle aneurysm, acute myocardial infarction, severe heart valve diseases, myocarditis, endocarditis, severe heart failure), and severe coagulopathy. Patients with two or more competing possible causes of stroke or unexamined patients were not included. Thus, the study group can be correspond to the subcategory of ischemic cryptogenic stroke, also called an ESUS [10]. In order to balance the study and the control groups for age and sex, the propensity score matching (PSM) was performed. This approach allowed us to reduce confounding biases in our observational study [22]. After PSM, 112 patients with ESUS and 47 healthy volunteers selected for the further analysis.

### 2.2. Data Collection

All study participants from both groups underwent the collection of baseline information (age, sex, body mass index), history of smoking, chronic diseases, and medication intake. The clinical examination was performed by a neurologist. The cognitive functioning was evaluated according to the Montreal Cognitive Assessment (MoCA) scale [23], and stroke severity and neurological deficit were estimated according to modified Rankin scale [24] and National Institutes of Health Stroke Scale (NIHSS) [25]. The instrumental examination included: electrocardiography (ECG), Holter 24-hr ECG monitoring, transthoracic echocardiography, extracranial brachiocephalic arteries high-resolution duplex scanning, transcranial cerebral arteries duplex scanning, and MRI of brain (Discovery MR 750w 3.0T, GE, USA).

### 2.3. Statistical Analysis

For the PSM, the R statistical software version 4.1.0. was used with the “MatchIt” package. The matching method was the nearest neighbor matching without replacement with the possibility of several experimental cases selection per control. As shown in Table 1, the PSM was carried out successfully and there were no statistically significant differences between the control and the study groups by sex (*p*=0.093) and age (*p*=0.079). Thereby, 47 cases for the control group and 112 cases for the study group (patients with ESUS) were selected for the further analysis.

Statistical analysis was performed using the SPSS Statistics version 23.0 (IBM) and R software version 4.1.0. The null hypothesis was rejected at a significance level of *p* < 0.05. Descriptive statistics was reported as frequency and percent for nominal variables and mean ± standard deviation for scale variables. In the case of ordinal variables and scale variables with a distribution that did not correspond to the normal, the median and quartiles were used. The normality of the scale variables' distribution was assessed using frequency histograms and the Kolmogorov–Smirnov test. For nominal-dependent variables, comparisons of frequencies between categories of independent (grouping) variables were performed using Pearson's *χ*^2^ test or Fisher's exact test. For scale-dependent variables, comparisons were made using the Student's *t*-test or (if the distribution of the variable did not correspond to normal) the Mann–Whitney *U* test. In all cases, we applied the multiplicity correction with the Bonferroni method. After the selection of ESUS potential predictors by the methods described above, a multivariate analysis was performed using the binary logistic regression method.

## 3. Results

**Figure 1:**
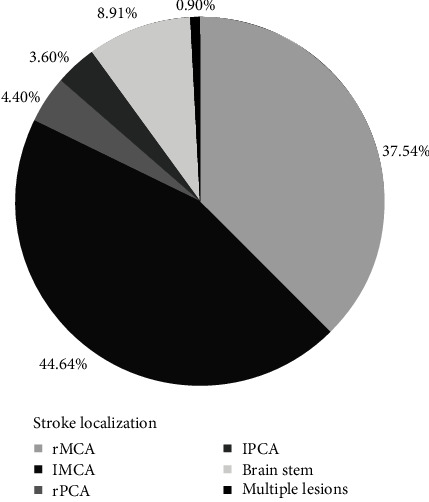
The diagram of the localization of ischemic lesions.

We analyzed the localization of ischemic lesions in all studied cases (*n* = 112) and the results are shown in Figure 1. According to the results, in 82.1% (92 cases), stroke occurred in anterior cerebral circulation, among which the right middle cerebral artery (MCA) and the left MCA constituted 37.5% (42 cases) and 44.6% (50 cases), respectively. The posterior cerebral circulation was involved in 17% (19 cases), among which in 8.9% (10 cases), the infarction occurred in the brain stem and in 8% (nine cases) in posterior cerebral artery (PCA) (4.4%—in right PCA, 3.6%—in left PCA). There was also one case with multiple lesions in both anterior and posterior circulation systems.

We compared group of patients with ESUS and asymptomatic volunteers according to the main demographic and anthropometric parameters, stroke risk factors, comorbidities, and cognitive functioning according to the MoCA scale score. It is worth noting that groups were balanced for age and sex after the PSM. The main results of the univariate statistical analysis are shown in Table 2. It was shown that in the group of patients with ESUS, the frequency of arterial hypertension (AH) was significantly higher and MoCA scale score was lower (*p* < 0.05). No statistically significant difference was found in age, sex, body mass index of patients, smoking addiction, incidence of diabetes mellitus, chronic kidney disease, as well as some other comorbidities such as gout and varicose veins. Additionally, we analyzed the frequency of medication intake in both groups. Patients with ESUS more often took statins (41.1% vs. 10.6%, *p* < 0.0005), antiplatelet drugs (42.9% vs. 17%, *p*=0.002), and antihypertensive drugs that suppress the activity of renin–angiotensin–aldosterone system (43.8% vs. 23.4%, *p*=0.020) at the time of examination. It should be noted that the described differences can be explained by the prescription of the secondary stroke prevention treatment to the patients after the stroke onset. At the same time, the groups did not differ in the frequency of taking anticoagulants (1.8% vs. 0%, *p*=0.581), beta-blockers (17.9% vs. 19.1%, *p*=0.826), calcium channel blockers (13.4% vs. 12.8%, *p*=1.000), diuretics (13.4% vs. 10.6%, *p*=0.795), insulin (1.8% vs. 2.1%, *p*=1.000), and other antidiabetic drugs (7.1% vs. 10.6%, *p*=0.529).

The results of the instrumental examination of both groups were well analyzed by the univariate statistical analysis. The main data of ECG, echocardiography, and MRI are shown in Table 3. As shown in Table 3, patients with ESUS more often had a conduction disturbance (bundle branch blocks) and a higher heart rate according to ECG data. There were no cases of atrial fibrillation in patients from both groups. According to the transthoracic echocardiography results, patients with ESUS more often have higher pulmonary arterial systolic pressure (PASP), larger left ventricle end-systolic length, left and right atrium enlargement, and left ventricular hypertrophy. Regarding patients with left ventricular hypertrophy, the frequency of occurrence of its different subtypes (concentric and eccentric) did not differ between the groups. In both groups, the cases with concentric hypertrophy prevailed, whereas the eccentric subtype was observed only in a small number of cases (data are given in Table 3).

**Figure 2:**
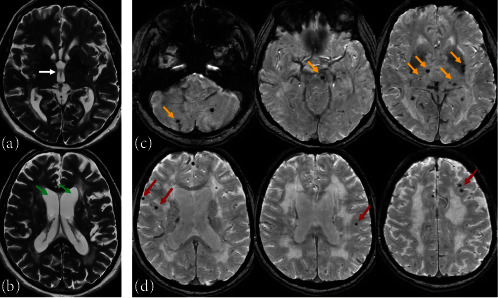
The examples of brain MRI finding of patients with ESUS. (a) T2 WI, white arrow indicates dilated third ventricle. (b) T2 WI, green arrows indicate the dilatation of the lateral ventricles due to central brain atrophy. (c) SWAN MRI, orange arrows indicate deep microbleeds. (d) SWAN MRI, red arrows indicate subcortical microbleeds.

Analysis of the MRI images revealed that patients with ESUS more often had microbleeds in subcortical regions. However, the frequency of microbleeds' occurrence in deep regions of the brain did not differ significantly between the groups. The severity and frequency of occurrence of periventricular and deep white matter lesions did not differ significantly between the groups as well. Patients with ESUS had significantly higher frequency of central brain atrophy occurrence, larger width of the third ventricle, and the higher medial temporal lobe atrophy (MTA) score compared to the control group. The examples of MRI findings are shown in Figure 2.

The results of high-resolution duplex ultrasound of the brachiocephalic arteries are shown in Table 4. As can be seen, in the group of patients with ESUS, the intima–media thickness (IMT) in both right and left common carotid artery (CCA) was significantly greater compared to the control group. At the same time, no differences were found in the atherosclerotic plaques' detection frequency and the degree of stenosis of the CCA and internal carotid artery (ICA).

At the next stage of our research, a multivariate analysis was performed using the binary logistic regression method in order to identify the association of the most prominent risk factor of ESUS and to generate the overall predictive model. The results of the binary logistic regression are given in Table 5. The binary logistic regression model was adjusted for age (*p*=0.53; OR = 0.99 (95% CI: 0.94–1.03)) and sex (*p*=0.046; OR for males = 2.77 (95% CI: 1.02–7.56)) since there was some tendency toward differences between groups for them. Based on the results of binary logistic regression, the following variables were selected in the final model: the presence of AH (*p* < 0.0005; OR = 12.98 (95% CI: 4.53–37.21)) and PASP (*p*=0.018; OR = 1.13 (95% CI: 1.02–1.25)). The Nagelkerke's pseudo-R-squared value was 0.404 and the significance of the Hosmer–Lemeshow test was 0.733, which indicate the goodness of the final logistic regression model. It should be noted that different cardiac function parameters are strongly correlated with each other; therefore, their simultaneous inclusion in the model may reduce its quality. Hence, among all cardiac function parameters, PASP had been chosen for the model as it gave the highest regression model accuracy.

**Figure 3:**
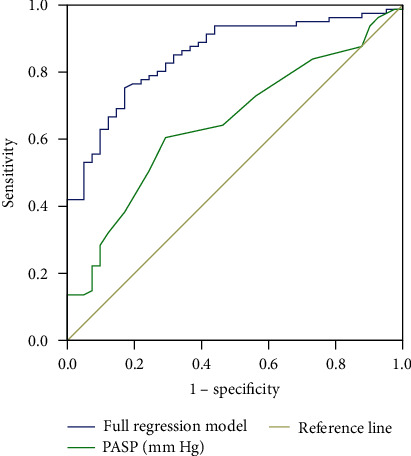
ROC curve for the model. The curves for the full logistic regression model and for the pulmonary arterial systolic pressure (PASP) are presented on the graph for the comparison.

The accuracy of the obtained binary logistic regression model was also assessed by the receiver operating characteristic (ROC) analysis. The ROC analysis was also performed for PASP for illustration purposes. The area under the curve was 0.853 (*p* < 0.0005 compared to the diagonal reference line), which corresponds to the good quality of the model's predictions. The results of ROC analysis are shown in Figure 3.

## 4. Discussion

In order to determine the most prominent risk factors and to generate the overall predictive model of ESUS, we conducted a hospital-based prospective cohort study. This exploratory analysis revealed the variety of potential predictors of ESUS: AH, factors associated with pathological changes in the heart (increased heart rate, conduction disturbance, left and right atrium enlargement, elevated PASP, and left ventricle end-systolic length), increased IMT in right and left CCA, signs of pathologic brain changes on MRI (subcortical microbleeds, central brain atrophy, the larger size of third ventricle, higher MTA score), and cognitive impairment (lowered MoСA scale score). The subsequent multivariate logistic analysis revealed the three main predictors of ESUS: the presence of AH, elevated PASP, and male sex. It should be noted that different cardiac function parameters are strongly interdependent [27, 28]; therefore, their simultaneous inclusion in the model may reduce its quality. Hence, among all cardiac function parameters, PASP had been chosen for the model, as it gave the highest regression model accuracy. If instead of PASP we include any other factor associated with heart changes (for example, heart rate or atrial enlargement) in the model, it would also be a significant predictor; however, the accuracy of the model would be worse. For example, Nagelkerke's pseudo-R-squared for the model with left atrium enlargement or heart rate would be 0.357 and 0.329, respectively.

By analyzing all revealed significant risk factors of ESUS described above, it can be concluded that the vast majority of them could be the consequences of AH. For instance, it was demonstrated that AH is strongly associated with small vessel diseases that clinically manifest as brain changes (cerebral microbleeds, brain atrophy, and the others) and cognitive deterioration that were found in our research [29–32]. It is also well known that AH may be the leading cause (however, not the only one) of the increased heart rate [33, 34], conduction disturbance, elevated PASP [35], and heart atrial and ventricular remodeling [36–38], all of which were identified in ESUS patients in the current study. It can be assumed that described mild/moderate pathological changes in the heart, especially remodeling of the chambers, may be the potential causes of the blood clots formation in ESUS patients. Obtained results are in agreement with the literature data. It was previously shown that left ventricular changes and its subsequent dysfunction, including the presence of wall motion abnormalities, may increase the likelihood of thrombus formation [39–42]. Recent study of Yoshida et al. [43] demonstrated that even the presence of subclinical left ventricular systolic dysfunction without severe heart failure is also a strong predictor of ischemic stroke incidence in the elderly population. Moreover, there is also growing evidence that blood clots may form in the enlarged left atrium even at the initial phase of myocardial remodeling long before the development of atrial fibrillation with blood flow slowing. The possible pathophysiological mechanisms in the remodeled atrial myocardium that leads to thrombogenesis are well reviewed by Sajeev et al. [44]. Ning et al. [45] also reviewed the data about the mechanisms and clinical evidence that suggested that atrial cardiomyopathy may be a potential mechanism of ESUS. The results of several other studies supported the described concept that atrial enlargement and cardiomyopathy can be associated with ESUS [46–49]. As it was mentioned above, randomized control trials failed to prove the hypothesis that the use of anticoagulants (rivaroxaban and dabigatran) can be more effective than antiplatelet therapy (aspirin) for the secondary stroke prevention in ESUS patients [46]. The reason for the neutral results is still unclear but probably can be associated with the heterogeneity of ESUS causes [26]. Thereby, the clinical trial aimed to identify groups of patients with ESUS who may benefit from anticoagulant therapy was initiated. In the ongoing ARCADIA trial, it was hypothesized that in the group of ESUS patients with atrial cardiopathy, apixaban can be superior to aspirin for the prevention of recurrent stroke [50]. The promising results of the clinical research are expected.

The second competing cause of ESUS is considered to be the nonstenotic large artery atherosclerosis according to the results of numerous studies [26, 49, 51–55]. The results of our study are in accordance with the literature data. We also found some tendency toward a greater severity of atherosclerosis in patients with ESUS; however, it was less pronounced than cardiac consequences of AH in our cohort of patients. By analyzing the degree of atherosclerosis of the large arteries, only the elevated IMT in right and left CCA differed significantly in ESUS patients compared to the healthy volunteers. It should be noted that increased carotid IMT, as the early sign of atherosclerosis [56], is also strongly associated with AH [57–60].

The results of our study indicate that AH and its consequences are the key to pathogenesis of ESUS. AH has long been considered to be a prevalent modifiable risk factor of various cerebrovascular disease, including ischemic stroke [61–63]. However, in case of ESUS, the impact of central blood pressure has not been fully assessed in clinical trials. Recently, Han et al. [64] determined the high prognostic value of elevated central blood pressure in the occurrence of recurrent stroke, unfavorable outcomes, and mortality in ESUS patients. In this way, our findings together with the data obtained by Han et al. [64] emphasize the importance of AH control for primary and secondary prevention of ESUS.

The main limitation of this study is moderate sample size and data collection from the single hospital. However, the patients were admitted to the hospital from all country regions; thus, the data represent a heterogeneous population. Among the strengths of the present research are the comparison of the study group and the control group consisted of healthy volunteer. Besides, the groups were balanced for age and sex with PSM, so the study contains case–control elements, which increases the quality of statistical analysis. It is worth noting that in the vast majority of works dedicated to ESUS predictors, patients with ESUS were compared with patients who had stroke of some other determined cause [26]. Therefore, some risk factors that may be common to both study groups could be underestimated in such analysis.

## 5. Conclusions

In this study, we identified several significant risk factors of ESUS: AH, increased heart rate and PASP, the presence of conduction disturbances, left and right atrium enlargement, elevation of left ventricle end-systolic length, increased IMT in right and left CCA, lowered MoСA cognitive scale score, the presence of subcortical microbleeds, central brain atrophy, the larger size of third ventricle, and higher MTA score. The subsequent multivariate logistic analysis revealed the three main predictors of ESUS: the presence of AH, elevated PASP, and male sex. Therefore, we propose that AH and its consequences may play a key role in pathogenesis of ESUS and can be considered as important prognostic factors. This study emphases the importance of well-timed control of AH before the onset of its complications, especially heart remodeling, for primary prevention of ESUS.

## Figures and Tables

**Table 1:** The distribution of age and sex characteristics in the study and the control groups after the PSM.

		Asymptomatic volunteers (control group)			Patients with ESUS (study group)		
		Women	Men	Total	Women	Men	Total
Age (years)	Mean	57	53	55	64	56	58
	Maximum	75	76	76	82	76	82
	Minimum	34	23	23	35	21	21
	Standard deviation	12	16	14	12	11	12
	Number of participants	20	27	47	31	81	112

**Table 2:** Demographic, anthropometric parameters, stroke risk factors, comorbidities, and MoCA score scale of asymptomatic volunteers and patients with ESUS.

	Asymptomatic volunteers (control group), *n* = 47	Patients with ESUS (study group), *n* = 112	Significance (*р*)
Age (years) (mean ± SD)	55 ± 14	58 ± 12	0.079
Male sex (*n* (%))	27 (57.4%)	81 (72.3%)	0.093
Body mass index (kg/m^2^) (mean ± SD)	26.93 ± 4.97	27.65 ± 4.59	0.402
Smoking (*n* (%))	10 (21.3%)	32 (28.8%)	0.431
Arterial hypertension (*n* (%))	15 (31.9%)	91 (81.3%)	**<0.0005**
Diabetes mellitus (*n* (%))	5 (10.6%)	25 (22.3%)	0.119
Chronic kidney disease (*n* (%))	1 (2.1%)	5 (4.5%)	0.671
MoСA cognitive scale overall score (median [Q1, Q3])	27 [26, 28]	22 [13, 28]	**0.001**

*p* Values below the usual significance bound (0.05) are highlighted in bold i.e. significant ones.

**Table 3:** The main results of the univariate statistical analysis based on the instrumental examination data.

	Asymptomatic volunteers (control group)	Patients with ESUS (study group)	Significance (*р*)
Сonduction disturbance (bundle branch blocks, ECG) (*n* (%))	3 (6.5%), *N* = 46	25 (23.8%), *N* = 105	**0.012**
Heart rate (bpm) (ECG) (mean ± SD)	63.65 ± 10.5, *N* = 46	68.73 ± 12, *N* = 105	**0.014**
Left atrium enlargement (echocardiography) (*n* (%))	9 (22.0%), *N* = 41	45 (47.9%), *N* = 94	**0.005**
Right atrium enlargement (echocardiography) (*n* (%))	4 (10.0%), *N* = 40	32 (34.4%), *N* = 93	**0.003**
Left ventricular hypertrophy, all subtypes (echocardiography) (*n* (%))	8 (21.1%), *N* = 38	38 (60.3%), *N* = 63	**<0.0005**
Eccentric left ventricular hypertrophy (echocardiography) (*n* (%))	1 (12.5%), *N* = 8	2 (5.3%), *N* = 38	0.444
Pulmonary arterial systolic pressure (mm Hg) (echocardiography) (mean ± SD)	25.83 ± 5.21, *N* = 41	28.88 ± 6.59, *N* = 94	**0.002**
Left ventricle end-diastolic volume (ml) (echocardiography) (mean ± SD)	101 ± 17, *N* = 41	103 ± 23, *N* = 94	0.606
Left ventricle ejection fraction (%) (echocardiography) (mean ± SD)	63 ± 5, *N* = 41	64 ± 6, *N* = 94	0.363
Left ventricle end-systolic length (mm) (echocardiography) (mean ± SD)	28.98 ± 3.43, *N* = 41	30.73 ± 3.30, *N* = 94	**0.018**
Subcortical microbleeds absence (MRI)	45 (95,7%), *N* = 47	81 (72.3%), *N* = 112	**0.006**
Deep microbleeds absence (MRI)	42 (89.4%), *N* = 47	81 (72.3%), *N* = 112	0.101
Periventricular white matter lesions absence (MRI, Fazekas score 0)	30 (63.8%), *N* = 47	48 (42.9%), *N* = 112	0.088
Width of the third ventricle (MRI, mm)	5.02 ± 2.07, *N* = 47	7.57 ± 2.54, *N* = 112	**<0.0005**
Central atrophy signs absence (MRI)	39 (83.0%), *N* = 47	64 (57.1%), *N* = 112	**0.041**
Right medial temporal lobe atrophy (MRI, MTA score 0)	29 (61.7%), *N* = 47	40 (35.7%), *N* = 112	**0.028**
Left medial temporal lobe atrophy (MRI, MTA score 0)	29 (61.7%), *N* = 47	48 (42.9%), *N* = 112	**0.049**

*N*, overall patients number. Significant *p* values are highlighted in bold.

**Table 4:** Indicators of the brachiocephalic arteries atherosclerosis severity according to ultrasound data.

	Asymptomatic volunteers (control group)	Patients with ESUS (study group)	Significance (*р*)
Atherosclerotic plaques in right CCA (*n* (%))	15 (32.6%), *N* = 46	50 (47.6%), *N* = 105	0.086
Atherosclerotic plaques in left CCA (*n* (%))	16 (34.8%), *N* = 46	52 (49.5%), *N* = 105	0.094
Atherosclerotic plaques in right ICA (*n* (%))	14 (30.4%), *N* = 46	46 (44.7%), *N* = 103	0.102
Atherosclerotic plaques in left ICA (*n* (%))	15 (32.6%), *N* = 46	47 (45.2%), *N* = 104	0.125
Degree of right CCA stenosis (%) (median [Q1, Q3])	30 [25, 30], *N* = 15	30 [25, 40], *N* = 50	0.326
Degree of left CCA stenosis (%) (median [Q1, Q3])	30 [25, 30], *N* = 16	30 [25, 35], *N* = 52	0.388
Degree of right ICA stenosis (%) (median [Q1, Q3])	30 [25, 30], *N* = 14	33 [25, 40], *N* = 46	0.120
Degree of left ICA stenosis (%) (median [Q1, Q3])	25 [25, 30], *N* = 15	30 [25, 40], *N* = 47	0.276
IMT in right CCA (1–1.5 cm proximal to bifurcation on the posterior wall out of the bounds of atherosclerotic plaque) (mm) (mean ± SD)	0.79 ± 0.25,; *N* = 46	0.91 ± 0.22, *N* = 104	**0.008**
IMT in left CCA (1–1.5 cm proximal to bifurcation on the posterior wall out of the bounds of atherosclerotic plaque) (mm) (mean ± SD)	0.82 ± 0.36, *N* = 46	0.93 ± 0.25, *N* = 104	**0.042**

The degrees of stenosis are shown as median [Q1, Q3], the intima–media thickness (IMT)—as mean ± standard deviation. *N*, overall patients number. Significant *p* values are highlighted in bold.

**Table 5:** The results of binary logistic regression analysis.

	Significance (*р*)	OR	95% CI for OR	
			Lower	Upper
Sex (male)	0.046	2.771	1.017	7.555
AH diagnosis	<0.0005	12.982	4.529	37.212
Age	0.530	0.985	0.941	1.032
PASP	0.018	1.132	1.021	1.254
Constant	0.003	0.017		

In the table, the dependent variable is the ESUS presence, and the independent variables included in the equation are presented in rows.

## Data Availability

The data presented in this study are available on request from the corresponding author.
